# Supplementary material for: Association between yoga and related contextual factors with moderate-to-vigorous physical activity among children and youth aged 5 to 17 years across five Indian states
Source: PeerJ. 2024 May 31;12:e17369. doi: 10.7717/peerj.17369 (PMC11146328; doi:10.7717/peerj.17369)
Supplement: Supplemental Information 2 [file peerj-12-17369-s002.docx]

**Table S2. Univariate regression analyses showing relationship between yoga practice, sociodemographic characteristics, contextual variables, and moderate to vigorous physical activity**

|  | **Standardized Beta** | **P-value** |
| --- | --- | --- |
| Average minutes of yoga practice per day | 0.604 (0.352, 0.856) | <0.001 |
| Age | 1.340 (-0.230, 2.910) | 0.095 |
| Gender - Female (Ref) |  |  |
| Male | 49.682 (40.493, 58.871) | <0.001 |
| Location - Rural (Ref) |  |  |
| Urban | 2.581 (-7.294, 12.456) | 0.609 |
| Has no active friends (Ref) |  |  |
| Has one or more active friends | 48.835 (30.383, 67.286) | <0.001 |
| School participates in sports competition with other schools – No (Ref) |  |  |
| Yes | 27.270 (11.836, 42.704) | <0.001 |
| School organizes physical activity – No (Ref) |  |  |
| Yes | 19.761 (9.792, 29.730) | <0.001 |
