# Supplementary material for: Association between yoga and related contextual factors with moderate-to-vigorous physical activity among children and youth aged 5 to 17 years across five Indian states
Source: PeerJ. 2024 May 31;12:e17369. doi: 10.7717/peerj.17369 (PMC11146328; doi:10.7717/peerj.17369)
Supplement: Supplemental Information 3 [file peerj-12-17369-s003.pdf]

Hirabai Cowasji Jehangir Medical Research Institute
Lower Ground Floor, Block V, Jehangir Hospital, 32 Sassoon Road, Pune 411001. India
Tel. No. : +91 20-26141340 (020-66811000, 66819999 Ext.: 1741 / 1633)
Email : hcjmri.jehangirpune@gmail.com **Website :** www.hcjmri.org.in

MEDICAL RESEARCH INSTITUTE

6th Jan 2021

Respected Sir/ Madam,

We are a team of researchers at H.C. Jehangir Medical Research Institute, Jehangir Hospital, Pune, Maharashtra.

We have extensive work in the field of children's health and nutrition. Through the last decade, it has been observed that physical activity levels among children have reduced steadily. To understand the reasons behind this reduction and explore if a policy change can be sought by our findings, we request you to please complete the Physical Activity survey form and help us help children lead an active life. We are approaching schools to conduct this survey since schools have a major role to play in the overall development of children as children spend most of their time there.

This survey consists of 2 forms:

The 1st is for the Principals as they are important stakeholders, and their perspective will help us understand the challenges to students being physically active.

The 2nd form is for the students which will help us understand their perspective and their present activity levels.

Your participation will greatly contribute towards improving the health of India's future generations.

We kindly request you to fill the survey form.

Yours sincerely,

Dr. Anuradha Khadilkar,
Deputy Director and Consultant Paediatrician,
Hirabai Cowasji Jehangir Medical Research Institute,
Jehangir Hospital, Pune, Maharashtra.
